# Analgesic, Anxiolytic and Anaesthetic Effects of Melatonin: New Potential Uses in Pediatrics

**DOI:** 10.3390/ijms16011209

**Published:** 2015-01-06

**Authors:** Lucia Marseglia, Gabriella D’Angelo, Sara Manti, Salvatore Aversa, Teresa Arrigo, Russel J. Reiter, Eloisa Gitto

**Affiliations:** 1 Neonatal and Paediatric Intensive Care Unit, Department of Paediatrics, University of Messina, Via Consolare Valeria, Messina 98125, Italy; E-Mails: gabridangelo@alice.it (G.D.); aversalvo@hotmail.com (S.A.); egitto@unime.it (E.G.); 2 Unit of Paediatric Genetics and Immunology, Department of Paediatrics, University of Messina, Via Consolare Valeria 1, Messina 98125, Italy; E-Mails: saramanti@hotmail.it (S.M.); tarrigo@unime.it (T.A.); 3 Department of Cellular and Structural Biology, University of Texas Health Science Center at San Antonio, San Antonio, TX 40729, USA; E-Mail: reiter@uthscsa.edu

**Keywords:** melatonin, pain, anxiety, premedication, anaesthesia, children

## Abstract

Exogenous melatonin is used in a number of situations, first and foremost in the treatment of sleep disorders and jet leg. However, the hypnotic, antinociceptive, and anticonvulsant properties of melatonin endow this neurohormone with the profile of a drug that modulates effects of anesthetic agents, supporting its potential use at different stages during anesthetic procedures, in both adults and children. In light of these properties, melatonin has been administered to children undergoing diagnostic procedures requiring sedation or general anesthesia, such as magnetic resonance imaging, auditory brainstem response tests and electroencephalogram. Controversial data support the use of melatonin as anxiolytic and antinociceptive agents in pediatric patients undergoing surgery. The aim of this review was to evaluate available evidence relating to efficacy and safety of melatonin as an analgesic and as a sedative agent in children. Melatonin and its analogs may have a role in antinociceptive therapies and as an alternative to midazolam in premedication of adults and children, although its effectiveness is still controversial and available data are clearly incomplete.

## 1. Introduction

Melatonin is an endogenous indoleamine produced and secreted by the pineal gland. It has recently been recognized as a “ubiquitously distributed and functionally diverse molecule” [1]. In fact, melatonin has several important physiological functions, including regulation of the circadian rhythms, modulation of season changes, in reproduction as well as antioxidant, oncostatic, anti-inflammatory and anticonvulsant effects [1]. Exogenous melatonin has a number of beneficial actions, first and foremost is its use in the treatment of sleep disorders and jet leg [2]. In addition to sleep promotion, melatonin exerts numerous other sedative and anti-excitatory effects that clearly go beyond sleep induction since they are also observed in nocturnally-active animals [3]. This has been frequently studied in relation to its anticonvulsant actions [4,5,6,7,8], which have been linked to a facilitating role of melatonin on γ-aminobutyric acid (GABA) transmission [9]. Experimental data support the analgesic and sedative role of melatonin [10,11]. In adult human, its analgesic role has been employed for treatment of diseases with chronic pain [12,13,14]. The hypnotic property of melatonin supports its possible use in different stages during anesthetic procedures, from premedication to induction of general anesthesia for the modulating effects of melatonin on anesthesia drugs [15].

For this purpose, melatonin can also be administered sublingually or orally in adults and children. The bioavailability of orally administered melatonin is reduced by a hepatic first pass effect. After its oral administration, a peak serum melatonin concentration is reached after approximately 60 min; thereafter its concentration declines over a four-hour period [16]. In numerous long-term studies in children and adults, no substantive side effects have been determined after its oral administration [17,18].

The aim of this review was to evaluate the available evidence related to the efficacy and safety of melatonin as an analgesic and sedative agent in pediatric patients.

## 2. Melatonin and Pain

Adequate pain control is a fundamental right of every patient, especially children [19]. Neonates are more sensitive to pain than older infants and adults [20,21] and the exposure to painful procedures have short- [22] and long-term consequences [23].

Among that broad range of effects attributed to melatonin, its role in analgesia, although mainly based on experimental data, emerges as being important because of its clinical implications. The physiologic mechanisms underlying the analgesic actions of melatonin have not been completely clarified and a variety of mechanisms have been proposed: β-endorphins, via GABA receptor involvement, opioid l-receptors and the nitric oxide (NO)-arginine pathway [5,6,7,8,9,10]. Some evidence suggests that melatonin enhances the release of β-endorphin from pituitary gland, while naloxone could antagonize the melatonin induced-nociceptive effects by simply blocking the binding of β-endorphin to opioid receptors [10]. Melatonin interacts with multiple receptor sites including opioidergic, benzodiazepinergic, muscarinic, nicotinic, serotonergic, α1-adrenergic, α2-adrenergic, and most importantly MT1/MT2 melatonergic receptors present in the dorsal horn of the spinal cord as well in the central nervous system [24]. Also relevant is the finding that the long-lasting analgesia induced by melatonin can be blocked by naloxone, suggesting that opioid receptors are involved in actions of melatonin [25].

The antinociceptive effect of melatonin may be linked to Gi-coupled melatonin receptors, to Gi-coupled opioid-l-receptors or GABA receptors with unknown downstream changes with a consequential reduction in anxiety and pain. GABA-B receptors, opiate receptors and melatonin membrane receptors are in fact Gi-protein coupled and reduce the concentration of the second messenger cAMP upon stimulation [26]. GABA-B receptor agonists have been shown to have analgesic properties [27]. Thus, it could be that at the molecular/cellular level, melatonin functions in the modulation of opioids and GABA-B receptors via presently unknown intracellular changes downstream of the common second messenger, cAMP. Also, the repeated administration of melatonin improves sleep and thereby may reduce anxiety, which leads to lower levels of pain [28].

In inflammatory pain, the mechanism of melatonin analgesia appears to be by inhibition of NO production, reducing activation of the transcription factor κB (NF-κB), the expression of cyclooxygenase and prostaglandins, and the recruitment of polymorphonuclear cells to the site of inflammation [29]. After the first report of melatonin effects on pain published in 1969 [30], several studies in mice [31,32], hamsters [33], rats [34], and human [35] showed that there is a circadian rhythm in pain perception. While such day–night rhythm of nociceptive sensitivity is suspected to be related to changes in opioid activity [36], the pineal gland has also been implicated in the modulation of the diurnal rhythm of nociception. Has been showed that during darkness, when plasma melatonin levels are highest, animals are less sensitive to nociception, and more sensitive to morphine [37,38]. In fact, using two different methods of nociceptive sensitivity tests (tall-flick test and hot-plate test), Bar-Or and Brown [39] have demonstrated that in rats maintained on a 12 h daily photoperiod, pain sensitivity was the lowest when tested 2 h before lights were on (−2 h) and highest 4 h into the photophase (+4 h). Following surgical pinealectomy or light-induced functional pinealectomy, the magnitude of difference in hot-plate response latencies between the two time points (*i.e.*, −2 *vs.* +4 h) was found to be reduced [39], implying that melatonin could be involved in modulating the diurnal rhythm of nociception in rats. Oral melatonin replacement at a physiological dose reproduces the baseline 24 h rhythm of urinary 6-sulphatoxymelatonin output and restores the rhythm in both pressure-induced and heat-mediated nociception that is abolished by light exposure [40]. Thus, evidence exists that there is a physiologic antinociceptive effect of melatonin driving a circadian rhythm in pain perception.

The analgesic action of melatonin has been demonstrated exclusively in the management of chronic pain [12,13,14] and no date are available about its use as post-operative analgesic. In fact, disturbances in melatonin secretion have been proposed to be part of the pathophysiology leading to the pain of fibromyalgia, when given at doses of 3 mg orally for 4 weeks, 30 min before sleeping time, melatonin significantly improved sleep quality and resulted in a reduction in the number of painful trigger points [12]. Administration of melatonin 3 mg for two weeks significantly attenuated abdominal pain and bloating, while reducing rectal pain sensitivity in adult patients with inflammatory bowel syndrome [13]. The urinary melatonin concentration was found to be depressed in subjects suffering from migraine and one trial showed that melatonin may have both therapeutic and a prophylactic benefit in adult patients suffering from migraine headaches [14].

Data are lacking on the use of melatonin for analgesic purposes in children and neonates.

Gitto and co-workers [41] hypothesized that melatonin may have beneficial effects as an analgesic antioxidant in preterm newborns that are subject to painful procedures, such as endotracheal intubation and mechanical ventilation. They evaluated the analgesic activity of melatonin using the Neonatal Infant Pain Scale (NIPS) before, during, and after 5 min of elective endotracheal intubation, and the Premature Infant Pain Profile (PIPP) score at 12, 24, 48, and 72 h during ventilation in two groups of premature neonates. In the former group 10 mg/kg of melatonin were intravenous administrated prior to intubation in addition to the standard procedural analgosedation treatment (fentanyl); the latter group only received standard analgosedative therapy. The levels of the pro- and anti-inflammatory cytokines (IL-6, IL-8, IL-10, and IL-12) implicated in pain response were evaluated in both groups. The pain score was similar in both groups at an early phase (NIPS), while it was lower in melatonin-treated infants at a late phase (PIPP score). Pro-inflammatory and anti-inflammatory cytokines were higher in the common sedation and analgesia group than in melatonin-treated infants suggesting the use of melatonin as an adjunct analgesic therapy during procedural pain, especially when an inflammatory component is involved.

## 3. Melatonin and Anxiety

The positive relationship between anxiety and pain is a common experience in the clinical settings [42] and it is known that pain-related anxiety can increase perceived pain intensity [43]. Pain and anxiety are often related in surgery, and in association with environmental changes such as a hospital stay, medication, stress, or general anesthesia can affect the sleep–wake cycle. Plasma melatonin levels, which play an important role in the regulation of sleep–wake cycles, are decreased after surgery and in hospitalized patients [44].

In these patients, melatonin, administered orally, has a number of benefits including facilitating the onset and the quality of sleep [45], regulating circadian rhythms and alleviating preoperative anxiety [46]. Melatonin and the non-selective MT1/MT2 receptor agonist, agomelatine, display anxiolytic-like properties in classical animal paradigms of anxiety [47,48,49].

It has also been demonstrated that the anxiolytic effect of melatonin occurs following the activation of the GABAergic system [50]. Melatonin produces a marked dose-dependent increases in GABA concentrations in the central nervous system [15]. This property of melatonin is likely to result from the mutual interaction between GABA and MT2 receptor systems [50]. In adults, several studies demonstrated that 5 mg oral melatonin given preoperatively had a clinical significant effect on anxiolysis [51] and improved the control of post-operative pain.

In the pediatric population, melatonin has been successfully used in the treatment of anxiety associated to sleep disorders [2], while data on lowering of preoperative anxious status are discordant. In fact, for Samarkandi and co-workers [52] the administration of melatonin or midazolam, each in doses of 0.25 or 0.5 mg/kg, was equally effective in alleviating anxiety in children undergoing surgery. Conversely, other studies did not confirm this result. In fact, oral melatonin at a dose of 0.05–0.4 mg/kg was less efficacious than midazolam (0.5 mg/kg) in reducing children anxiety [53] and in a comparative study conducted on children undergoing sedation for dental treatments, who received either 3 mg melatonin *vs.* midazolam (15 mg), melatonin did not reduce the anxious status [54].

## 4. Melatonin and Anaesthetic Procedures

The sedative and hypnotic effects of melatonin [11] support its possible use in anesthetic procedures, from premedication to induction of general anesthesia [15].

### 4.1. Premedication

Midazolam was first introduced as a premedication for children in the 1980s and rapidly achieved widespread acceptance as the preferred premedication before induction of anesthesia. Although it can be administered via multiple routes, it is currently preferred as oral medication because of its rapid absorption and low incidence of nausea *vs.* other benzodiazepines [55]. However, midazolam has several side effects, including paradoxical reactions, interactions with opioids, variable bioavailability and elimination half-life, excessive sedation, disorientation, impaired psychomotor performance, and amnesia [56]. In light of these findings, melatonin has been proposed as alternative to midazolam as a premedicant in procedures preceding anesthesia induction.

A systematic review of recent literature [51,52,53,57] confirmed the controversial nature of the data on efficacy of perioperative melatonin in pediatric patients. In the aforementioned study of Samarkandi and colleagues [52] melatonin was not only as effective as midazolam in alleviating preoperative anxiety in children, but it was associated with a tendency toward faster recovery, lower incidence of excitement at 10 min postoperatively and of sleep disturbance at week two postoperatively than that observed with midazolam. On the contrary, in another study, has been demonstrated that midazolam was more effective than melatonin in reducing children’s anxiety at induction of anesthesia, but the individuals who received melatonin developed less emergence of delirium compared to those who received midazolam [53].

### 4.2. Sedation

General anesthesia, a pharmacologically-induced state that entails amnesia, analgesia, hypnosis, and, immobility, can be induced by a variety of intravenous and inhalational agents acting at different target sites [58]. At the molecular level, general anesthetics enhance the function of inhibitory *c*-aminobutyric acid type A (GABAA) and glycine receptors and inhibit excitatory nicotinic acetylcholine, serotonin type 3, and *N*-methyld- aspartate (NMDA) receptors [59,60,61].

It has been demonstrated that melatonin administration produces significant, dose-dependent, increases in GABA concentrations in the central nervous system.

Possible utility of high-dose intravenous (IV) melatonin as an anesthetic adjuvant has been studied. Data from *in vivo* rat models have shown that both melatonin and the more potent melatonin analogs, 2-bromomelatonin and phenylmelatonin, posses anesthetic properties, with the added advantage of providing potent antinociceptive effects [62,63]. Another study examined whether melatonin, at doses greater than those that produce somnolence, had properties considered beneficial in anesthetic adjuvants or in general anesthetics *per se* [64]. Intravenous melatonin exerted a profound dose-dependent hypnotic state in rats that was characterized by a rapid loss of righting reflex. However, the antinociceptive action of melatonin was considerably less potent than either propofol or thiopental, as melatonin was not effective in abolishing the response to tail clamping [64]. Thus, while melatonin appears to have properties of interest in an anesthetic adjuvant, it does not itself possess sufficient efficacy to warrant consideration as a general anesthetic.

In light of these properties, melatonin has been administered to children undergoing diagnostic procedures that required sedation or general anesthesia. In a survey on 250 children who underwent auditory brainstem response tests, oral melatonin (from 5 to 20 mg depending on age) induced which sleep allowed for the completion of the exploration in 74%–87% of cases [65]. The use of melatonin, especially in children younger than three years, has decreased significantly the number of youngsters undergoing general anesthesia for this diagnostic exploration. Widely accepted by parents, it permitted earlier diagnosis and treatment of hearing loss in children [65].

Magnetic resonance imaging (MRI) examination, to ensure immobility in children who are uncooperative, also requires sedation or general anesthesia. Both strategies are effective but can have side effects, carry a small mortality risk and require appreciable manpower resources. Natural sleep is an attractive option provided it is predictable and reliable, but at present, it is practical only in small infants who sleep after a feed [66]. Two studies have suggested that melatonin alone is useful for children having an MRI. Wassmer and colleagues [67] used melatonin at doses of 0.25–0.5 mg/kg in 27 children of whom 16 were scanned successfully. Johnson and colleagues [68] reported that 55% of children who received 10 mg melatonin were sufficiently asleep to compete the scan; the success rate increased to 76% if children had been sleep deprived. Whereas these results suggest that melatonin, at a higher dose, may have a sleep-promoting effect, neither study was randomized and patients who were “easy to sedate” may have been unintentionally selected. In general, in this field data are inconsistent. A stratified randomized double-blind study carried out on 98 pediatric patients treated with either melatonin (3–6 mg) or placebo, 10 min before sedation with chloral hydrate or temazepam plus droperidol for MRI examination reported that melatonin did not contribute to sedation [69]. Finally, the electroencephalography (EEG) procedure is not well tolerated by children, especially with developmental disabilities, and is preferentially recorded during sleep, particularly in the earlier stages of life. In children sleep EEG procedure, when properly performed, increases the amount of information on brain maturation and electrical activity and minimizes the presence of artifacts due to the relative lack of co-operation. Sleep is usually achieved either by partial deprivation or by the administration of sleep-inducing medications [70]. Wassmer and co-workers [71] used melatonin (2.5 or 5 mg depending on the age) in 68 selected children for sleep EEG. When compared to sleep deprivation, which is the standard procedure for obtaining sleep in pediatric EEG laboratories, melatonin administration at the dose previously mentioned promoted sleep during EEG without significant differences in sleep macrostructure, with the exception of shorter sleep latency. They concluded that melatonin is useful in children to induce sleep during EEG and that it is a potential alternative to pharmacological sedation.

Ashrafi and co-workers [72], in a study carried out on 348 patients, compared melatonin with chloral hydrate for recording sleep EEG. Patients, partially sleep-deprived the night before, were randomly divided in two groups and given melatonin and chloral hydrate on an alternative day basis, 174 patients in each group. The authors affirmed that sleep duration and drowsiness time were significantly shorter in the melatonin-treated children compared to those who received chloral hydrate. The authors concluded that melatonin was a plausible alternative to chloral hydrate for sleep induction without significant adverse effects for recording of sleep EEGs in the pediatric population, although the absence of a control group is a critical limitation. Further work is needed to better understand the general anesthetic properties of melatonin at the molecular, cellular, and systemic levels.

## 5. Conclusions

Melatonin, based on its properties as antioxidant, or analgesic, all of which have been confirmed in numerous experimental studies, offers perspectives of beneficial effects in a variety of pediatric therapies. Melatonin has been shown to exert antinociceptive actions in a variety of experimental animal models and in humans, suggesting that melatonin and its analogs may have a role in antinociceptive therapies.

Melatonin premedication has been used as an alternative to midazolam in adults and children, although its effectiveness is still controversial and available data are clearly incomplete.

Data also support the notion that melatonin, or one of its analogs, might find use as an anesthetic agent or adjuvant. These properties of melatonin have been used in diagnostic situations requiring sedation. Even if the intravenous administration of melatonin exerts hypnotic effects, it seems not to possess sufficient efficacy to warrant consideration as a general anesthetic. However, oral premedication with melatonin significantly reduces the propofol and thiopental doses required for sedation [73] and it may be used as a general anesthetic adjuvant.

Equally important to point out is that significant short- and long-term side effects after administration of standard and high doses (20 mg) of melatonin in children have not been reported [74,75,76,77,78].

However, the number of controlled clinical trials is still small, and specific studies to resolve problems of dosage, formulations, and length of treatment are desirable.
